# Association of red blood cell transfusion and in-hospital mortality in patients admitted to the intensive care unit: a systematic review and meta-analysis

**DOI:** 10.1186/s13054-014-0515-z

**Published:** 2014-11-14

**Authors:** Yi Zheng, Caihong Lu, Shiqing Wei, Ye Li, Lu Long, Ping Yin

**Affiliations:** Department of Epidemiology and Biostatistics, School of Public Health, Tongji Medical College, Huazhong University of Science and Technology, 1037 Luoyu Road, Hongshan, Wuhan, China; Department of ENT department, Union Hospital, Tongji Medical College, Huazhong University of Science and Technology, 1037 Luoyu Road, Hongshan, Wuhan, China

## Abstract

**Introduction:**

Previous research has debated whether red blood cell (RBC) transfusion is associated with decreased or increased mortality in patients admitted to the intensive care unit (ICU). We conducted a systematic review and meta-analysis to assess the relationship of RBC transfusion with in-hospital mortality in ICU patients.

**Methods:**

We carried out a literature search on Medline (1950 through May 2013), Web of Science (1986 through May 2013) and Embase (1980 through May 2013). We included all prospective and retrospective studies on the association between RBC transfusion and in-hospital mortality in ICU patients. The relative risk for the overall pooled effects was estimated by random effects model. Sensitivity analyses were conducted to assess potential bias.

**Results:**

The meta-analysis included 28,797 participants from 18 studies. The pooled relative risk for transfused versus nontransfused ICU patients was 1.431 (95% CI, 1.105 to 1.854). In sensitivity analyses, the pooled relative risk was 1.211 (95% CI, 0.975 to 1.505) if excluding studies without adjustment for confounders, 1.178 (95% CI, 0.937 to 1.481) if excluding studies with relative high risk of bias, and 0.901 (95% CI, 0.622 to 1.305) if excluding studies without reporting hazard ratio (HR) or relative risk (RR) as an effect size measure. Subgroup analyses revealed increased risks in studies enrolling patients from all ICU admissions (RR 1.513, 95%CI 1.123 to 2.039), studies without reporting information on leukoreduction (RR 1.851, 95%CI 1.229 to 2.786), studies reporting unadjusted effect estimates (RR 3.933, 95%CI 2.107 to 7.343), and studies using odds ratio as an effect measure (RR 1.465, 95%CI 1.049 to 2.045). Meta-regression analyses showed that RBC transfusion could decrease risk of mortality in older patients (slope coefficient −0.0417, 95%CI −0.0680 to −0.0154).

**Conclusions:**

There is lack of strong evidence to support the notion that ICU patients who receive RBC transfusion have an increased risk of in-hospital death. In studies adjusted for confounders, we found that RBC transfusion does not increase the risk of in-hospital mortality in ICU patients. Type of patient, information on leukoreduction, statistical method, mean age of patient enrolled and publication year of the article may account for the disagreement between previous studies.

**Electronic supplementary material:**

The online version of this article (doi:10.1186/s13054-014-0515-z) contains supplementary material, which is available to authorized users.

## Introduction

Anemia is highly prevalent among critically ill patients [1]. Nearly 60% of the patients admitted to intensive care units (ICUs) had a baseline hemoglobin (Hb) level less than 12 g/dL and 30% less than 9 g/dL [2-4]. Anemia persisted throughout the duration of their ICU stay and long after ICU discharge [5]. Anemia could lead to a decreased tissue oxygen delivery capacity, and is associated with poor outcomes, including acute myocardial infarction, heart failure, chronic kidney disease and risk of death [6-10].

Transfusion of red blood cells (RBCs) has been one of the most important treatments in clinical practice to improve tissue oxygenation. In the CRIT study, 44% of patients admitted to ICU received at least one RBC treatment and the mean time to first transfusion was 2.3 ± 3.7 days [3]. However, over the last two decades concerns have been raised that the possible risk of blood transfusion may outweigh the benefits. Studies in trauma, sepsis and acute respiratory distress syndrome (ARDS) patients reported a nonimprovement in tissue oxygen delivery [11-13], or even a decreased oxygenation through the storage and preparation of RBCs [14,15]. The use of allogeneic blood is also associated with potential adverse effects, including immunosuppression [16], risk for infection, transfusion reactions [17] and transfusion-related acute lung injury (ALI) (TRALI) [18]. Most seriously, blood transfusion may be associated with higher mortality [2,3,19]. A meta-analysis published in 2008 demonstrated an association between RBC transfusion and mortality in critically ill patients: 12 published studies were included, and the pooled odds ratio (OR) was 1.7 (95% confidence interval (CI), 1.4 to 1.9) [20]. However, this association has recently been questioned. Some studies reported that RBC transfusion was associated with a lower rate of mortality. Sakr *et al.* reported a decreased risk of in-hospital death in blood-transfused surgical ICU patients (relative risk (RR) 0.96, 95%CI 0.95 to 0.98), especially in patients aged from 66 to 80 years, in patients admitted to the ICU after noncardiovascular surgery, in patients with severe sepsis and in patients with high simplified acute physiology score II (SAPS II) or sepsis-related organ failure assessment (SOFA) scores [21]. More recently, Park *et al*. found a lower risk of in-hospital death in transfused patients with severe sepsis and septic shock (HR 0.51, 95%CI, 0.39 to 0.69) [4]. Despite all the controversy, clinical practice had been improved by the growing recognition of transfusion-related risks. Bilgin *et al*. and Hebert *et al*. had reported decreased mortality among patients receiving prestorage leukoreduced blood compared to patients receiving nonfiltered blood [22,23], indicating that leukocytes from donated blood may be crucial in the development of immune system suppression. Another transfusion practice is using ‘fresh’ transfused RBCs instead of ‘old’ blood; some studies suggested that the use of old stored RBCs is a potential risk factor for mortality [24,25]. There is also a transfusion practice for ICU patients considering a low transfusion threshold safer. A multicenter, randomized controlled trial (RCT) by Hebert *et al*., comparing liberal (Hb level, 10 to 12 g/dL) and restrictive (Hb level, less than 7 g/dL) RBC transfusion strategy, showed that the restrictive strategy could decrease rates of organ dysfunction, cardiac complications and mortality [26].

As research on the benefits and risks of RBC transfusion gains popularity, however, the results from previous studies remain confusing: is allogeneic RBC transfusion beneficial or harmful to ICU patients? And what kind of transfusion practices are effective in decreasing mortality? We thus conducted a meta-analysis of published retrospective and prospective observational studies comparing RBC transfused with nontransfused ICU patients to assess: (1) all-cause in-hospital mortality rate and (2) risk factors of death in transfused patient.

## Methods

### Data sources and searches

We followed the Strengthening the Reporting of Observational Studies in Epidemiology (STROBE) statement for observational studies [27]. We used a comprehensive search strategy to identify all potentially relevant studies by searching Medline, Web of Science and EMBASE. Databases were searched regardless of language and geography, and the published time restricted to 1980 through 31 May 2013. The search used terms (‘mortality’) AND (‘blood transfusion’ OR ‘anemia’ OR ‘erythrocyte transfusion’). To identify additional studies, reference lists of retrieved articles were also evaluated.

### Study selection

Two reviewers (YZ and CL) independently reviewed and extracted studies abided by the following inclusion criteria: (1) studies on adult subjects, or patients aged older than 16 years, (2) studies enrolling subjects from ICUs (medical or surgical), (3) studies dividing subjects into two groups according to whether they received transfusion or not, and reporting results focused on all-cause in-hospital mortality rate. For hospitalized ICU patients, in-hospital mortality is a short-term mortality, as Vincent *et al*. investigated that transfused patients admitted to ICU had a mean hospital length of stay (LOS) of 15.8 (9.0) days [2], and Sakr *et al*. found that the hospital LOS of surgical ICU patient was from 9 to 19 days, with a mean value of 12 days [21]. So our review was focused on in-hospital mortality of less than a month. Exclusion criteria were as follows: (1) studies on senior subjects, defined as aged more than 80 years, and (2) case reports or reviews.

All titles and abstracts reporting on blood transfusion and mortality were reviewed. All abstracts reporting on the association between blood transfusion and mortality or mobility were selected for full-text review.

### Data extraction

The primary outcome of interest was in-hospital mortality of transfused ICU patients comparing transfused (exposed group) versus nontransfused ICU patients. While extracting data, we found that except for ‘in-hospital mortality’ rate, some studies reported a ‘28-day mortality’ rate or ‘30-day mortality’ rate, in our review both were regarded as approximate ‘in-hospital mortality’ rate. In some of the studies, both adjusted results (OR, or RR, or hazard ratio (HR)) and count data (the numbers of survivors and nonsurvivors) were provided; or both propensity matched results and no propensity matched results were available. In these situations, we selected data for our meta-analysis complying with the following rules: (1) adjusted results had precedence over count data; (2) HR and RR estimates had precedence over OR estimates; (3) propensity matched results had precedence over no propensity matched results. The secondary outcomes were factors associated with transfusion status. We extracted relevant data from the literature as follows: (1) the type of reported effect size, (2) publication year, (3) adjustment for confounder, (4) mean admission Hb level, (5) percentage of patients transfused in ICU, (6) mean volume of transfused RBCs per patient, (7) mean pretransfusion Hb level, (8) mean admission APACHE II score, (9) leukodepleted blood measurement, (10) mean age of patients, and (11) type of patient.

### Study quality assessment

The Strengthening the Reporting of Observational Studies in Epidemiology (STROBE) statement for observational studies [27] was used to assess the quality of the included studies. The STROBE statement provided a checklist of six domains including title and abstract, introduction, methods, results, discussion and other information sections of articles to assess the quality of the observational studies. Each of these domains was evaluated as low or high risk of bias. Overall quality of the article was assessed as low (high risk of bias in more than five domains), median (high risk of bias in three or four domains), high (high risk of bias in one or two domains) and very high (high risk of bias in none of the domains). Only primary outcome (all-cause in-hospital mortality) was used to assess the risk of bias.

### Data synthesis and analysis

We used comprehensive meta-analysis software version 2.0 (Biostat, Englewood, NJ, USA) [28] to estimate pooled effect size. RR was chosen as the common measurement of association across studies, and a random effects model was used to generate a pooled RR estimate. HR was considered as RR directly. OR was transformed into RR if feasible. If effect estimates of mortality were not available from the original article, then the effect size was derived from the standard 2 × 2 table. The method used for transformation from OR to RR was according to Zhang *et al*. [29]:

1 $$ RR= OR/\left[\left(1-{P}_0\right)+\left({P}_0\times OR\right)\right] $$

And the standard error of logRR is:

2 $$ SE(logRR)=SE(logOR)\times logRR/ logOR $$

*P*_0_ represent the mortality rate in the nonexposed group.

When *P*_0_ was not available, then OR was directly considered as RR. Because the transformation can underestimate the variance of the RR derived from the OR [30], a sensitivity analysis was performed excluding studies that did not report adjusted RR or HR as the effect size. Another sensitivity analysis was performed excluding studies with low or median study quality. Meta-regression analyses using unrestricted maximum likelihood model were performed to assess the factors explaining the heterogeneity of risk ratios across studies. Stratified analyses were performed to assess the impact of transfusion status and mortality. Forest plots were constructed for selected studies, and *Q* statistic and *I*^2^ index were calculated to assess heterogeneity of outcome measures across studies. Begg’s rank correlation test and funnel plots were performed to detect publication bias through visual inspection. We assessed small study effect through the trim-and-fill method.

### Ethical approval

This study was reviewed and approved by the research ethics committee of School of Public Health, Tongji Medical College, Huazhong University of Science and Technology, Wuhan, China on 25 February 2013. Consent from patients was irrelevant in the present study (a systematic review).

## Results

### Study selection

**Figure 1:**
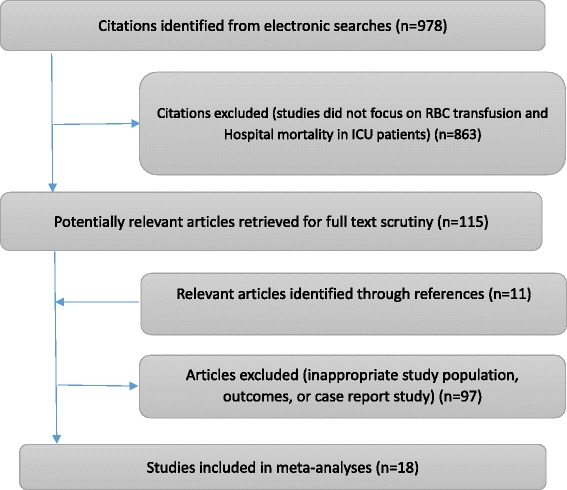
**Details of study selection for systematic review.**

The initial literature search yielded 978 studies (Figure 1). The titles and abstracts were examined independently by two authors to identify potentially relevant studies. After preliminary screening, 115 studies were left for full-text review. By searching potentially relevant articles from references, we further identified 11 studies for full-text review, but none of them met the inclusion criteria. Finally, 18 studies (28,797 participants) were identified as relevant to the investigation of mortality of transfused ICU patients, all of which were observational studies. Eight studies were prospective and the other ten retrospective. For one study [31], the authors reported an obvious wrong effect size, with the OR 2.46, and its 95%CI 3.17 to 11.56. We calculated the crude effect value through count data, and the crude OR (95%CI) was 5.33 (3.17 to 11.56). Table 1 provides detail information of the 18 included studies [2-4,19,21,31-43].

**Table 1:** **Details of studies included in meta-analysis (n = 18)**

**Study**	**Study designation**	**Inclusion population**	**Exclusion population**	**Adjustment for confounding**
Vincent *et al*. [2]	Prospective, multiple center, observational study in western Europe	All ICU patients	Not reported	Patients’ admission variables
Corwin *et al*. [3]	Prospective, multiple center, observational cohort study in the United States	Not reported	Admission to a pediatric, cardiothoracic, cardiac, neurologic, or burn ICU; renal failure on dialysis	Propensity to receive a transfusion
Robinson *et al*. [32]	Retrospective, observational study	Not reported	Patients with blunt hepatic, splenic, or both injuries	Shock indices and associated injury severity
Croce *et al*. [31]	Retrospective, observational study	Patients with blunt injury and ISS <25, survival of at least 48 hours, and no blood transfusion within the first 48 hours from admission	Patients who received any transfusion within the first 48 hours from admission, ISS > =25	We use original numbers to analyze RR, because the author reported a distracting result (OR2.46,95%CI, 3.17 to 11.56)
Taylor *et al*. [19]	Prospective, observational, cohort study	All ICU admissions	Not reported	Unadjusted
Netzer *et al*. [33]	Prospective, cohort study	Patients with ALI/ARDS	Patients were excluded if they had current or prior congestive heart failure, respiratory disease, or conditions that mimicked ALI/ARDS, including vasculitis with diffuse alveolar hemorrhage; were burned 30% of total body area; or were lung or bone marrow recipients.	Age, gender, APACHE III score, and precipitating event
Ruttinger *et al.* [34]	Retrospective, observational cohort study	All consecutive cases admitted immediately or delayed after a surgical procedure	Patients who had not undergone surgery during their present hospital stay and who had been admitted only for medical reasons, and patients with a rapidly fatal clinical course or with minimal disease severity	Admission variables, maximum APACHE II score, maximum number of failing organs, duration of invasive ventilation, duration of catecholamine therapy, and duration of renal replacement therapy
Vincent *et al*. [35]	Prospective, multicenter, observational study	All ICU patients	Not reported	Sex and age, type of admission, main medical history, fluid balance, SAPS II, and severity of organ dysfunction on admission as SOFA score
Bochicchio *et al*. [36]	Prospective	Trauma patients admitted >48 hours to the ICU	Not reported	Age, sex, race, and ISS
Bursi *et al*. [37]	Retrospective observational study	Stable patients after elective major vascular surgery	Patients who had hemorrhagic hypovolemic shock requiring emergency RBC transfusion, severely anemic	Baseline characteristics, surgical risk, bleeding, presence of anemia, and propensity to receive transfusion
Engoren *et al*. [38]	Retrospective study	All ICU patients	Cardiac surgical patients	APACHE II scores and propensity to receive a transfusion
Sakr *et al.* [21]	Retrospective study	All surgical ICU patients	Not reported	Patients’ propensity to receive a transfusion
Parsons *et al*. [39]	A secondary analysis	Patients with new-onset ALI, sepsis and shock	Patients with trauma or multiple transfusion	Age, sex, race, randomization arm and APACHE III score
Sheth *et al*. [40]	Retrospective, observational cohort study	Patients with intracerebral hemorrhage	Patients younger than 18 years of age or with ICH secondary to antecedent head trauma, acute ischemic stroke with hemorrhagic transformation, brain tumor, vascular malformation, venous thrombosis, vasculitis of the central nervous system, hematological malignances, blood dyscrasia, or coagulopathy	Anemia, warfarin use, admission GCS score, hematoma volume, hematoma location, and DNR/CMO status
Park *et al*. [4]	Prospective, multicenter observational study	Patients with severe sepsis or septic shock	Not reported	Propensity to receive a transfusion
Brophy *et al*. [41]	A cross-sectional retrospective study	Anemia and renal dysfunction	Patients with anemia of neoplastic diseases or those receiving chemotherapy	Age, race, sex, ICU LOS, ESA use, transfusion status, mechanical ventilation or CPAP status, vasopressor use, severity of, illness, and presence of, following comorbid conditions, GI bleed, sepsis, and neurologic injury.
Silva *et al.* [42]	Prospective observational cohort study	All ICU admissions	Acute coronary syndrome, ischemic stroke, acute hemorrhage, prior transfusion, pregnant women and Jehovah’s Witnesses	Sex, origin, previous disease, ventilation mode
Sekhon *et al*. [43]	Retrospective cohort study	Severe TBI patients	Nontraumatic etiology, consciousness, concomitant traumatic quadriparesis	Age, admission GCS score, insertion of EVD, mean 7-day hemoglobin

APACHE, acute physiology and chronic health evaluation; CPAP, continuous positive airway pressure; DNR/CMO, do not resuscitate/comfort measures only; ESA, erythropoiesis-stimulating agents; EVD, external ventricular drain; GCS, Glasgow coma score; GI, gastrointestinal; ICU, intensive care unit; LOS, length of stay; OR, odds ratio; RBC, red blood cells; RR, relative risk; SAPS, simplified acute physiology score; SOFA, sepsis-related organ failure assessment; TBI, traumatic brain injury.

### Risk of bias assessment

The risk of bias assessment is represented in Figure S1 in Additional file 1. We found that, title/abstract, introduction, results and discussion sections had a low risk of bias, other information had a median risk of bias and methods had a high risk of bias. Only one study did not indicate the study’s design clearly [31]. Two studies did not discuss limitations of the study and take any sources of potential bias or imprecision into account [31,32]. Analyses of subgroups were not reported in three studies [32,42,43]; eleven studies did not provide information on handling of missing data. Six studies reached very high overall quality [2,34,35,37,39,41], nine studies reached high overall quality [4,19,21,33,36,38,40], and the other three studies reached median overall quality [31,32,42], no study reached low overall quality. All risk of bias assessments were conducted by two authors (YZ and CL). Disagreements were resolved by consensus.

### Synthesis of results

**Figure 2:**
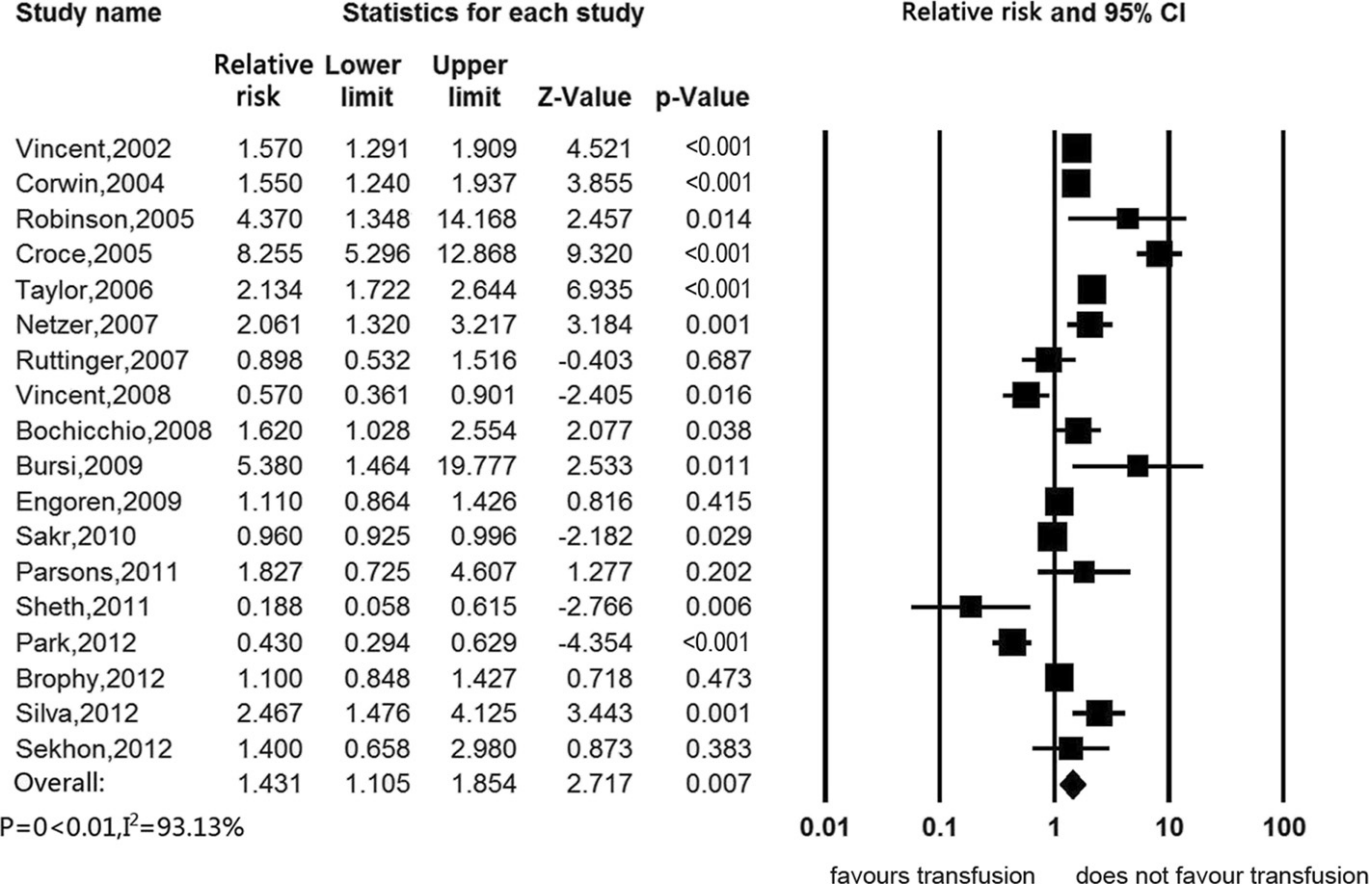
**Association between red blood cell transfusion and in-hospital mortality.**

The overall pooled risk ratio of in-hospital mortality of transfused patients compared to nontransfused was about 1.431 (95%CI, 1.105 to 1.854) (Figure 2).

**Figure 3:**
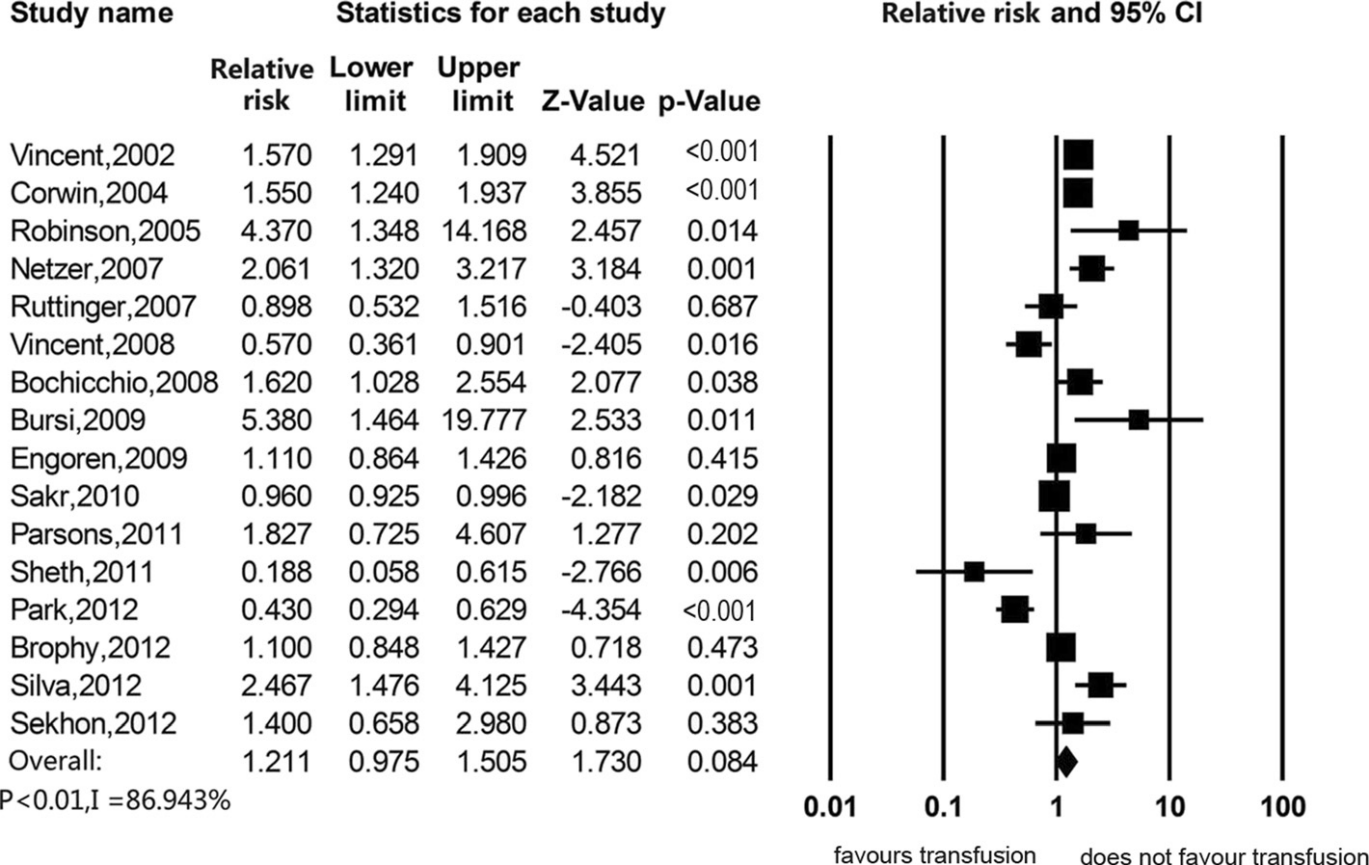
**Association between red blood cell transfusion and in-hospital mortality, excluding studies that did not use any adjustment for confounding.**

**Figure 4:**
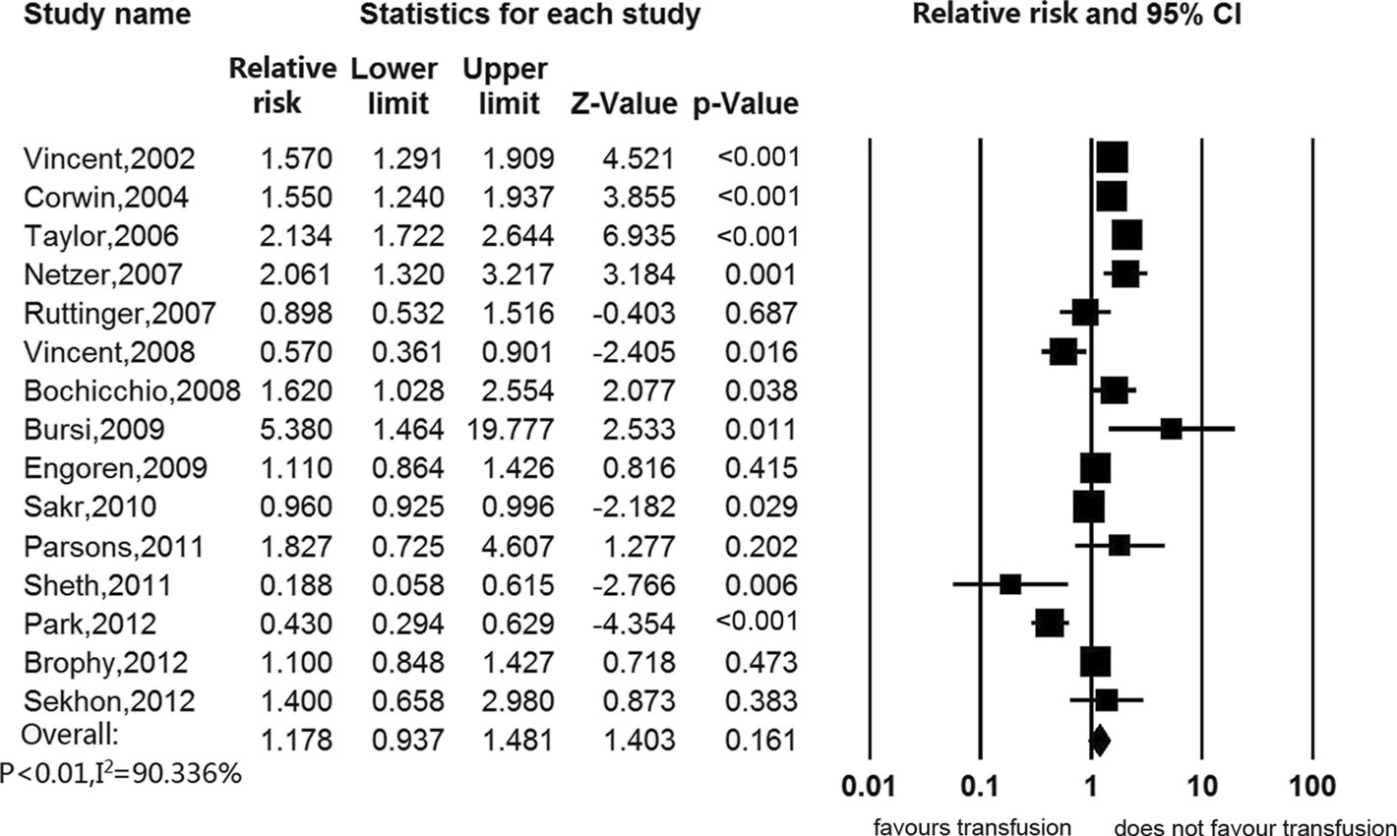
**Association between red blood cell transfusion and in-hospital mortality, excluding studies with a quality score as median or low quality.**

**Figure 5:**
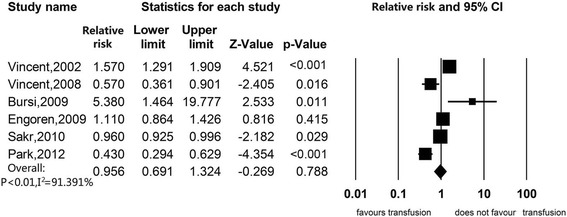
**Association between red blood cell transfusion and in-hospital mortality, excluding studies not reporting hazard ratio or relative risk as the effect size measurement.**

Sensitivity analyses were presented in Table 2. When excluding studies that did not adjust for any confounding factors, the estimated association of RBC transfusion with in-hospital mortality was null (RR 1.211, 95%CI, 0.975 to 1.505) (Figure 3). When we excluded studies with median or low quality, there was also no association (RR 1.178, 95%CI, 0.937 to 1.481) (Figure 4). When we excluded studies without reporting HR or RR as the effect size measurement, still there was no significant association (RR 0.901, 95%CI 0.622 to 1.305) (Figure 5). Subgroup analyses were stratified by type of study patients, type of reported effect size, confounder adjustment methods and leukodepleted blood measurement (Table 3) (Figure S2 in Additional file 2, Figure S3 in Additional file 3, Figure S4 in Additional file 4 and Figure S5 in Additional file 5). Results of subgroup analyses suggested that blood transfusion was a risk factor in studies enrolling patients from all ICU admissions (RR 1.513, 95%CI 1.123 to 2.039), studies that reported OR as the effect size (RR 1.465, 95%CI, 1.049 to 2.045), studies that did not adjust for any confounders (RR 3.933, 95%CI 2.107 to 7.343) and studies that did not provide available information on leukoreduction (RR 1.851, 95%CI, 1.229 to 2.786), while other subsets of studies suggested that RBC transfusion was not associated with mortality.

**Table 2:** **Details of sensitivity analysis of pooled risk ratio (95%CI) for in-hospital mortality**

	**Number of studies excluded**	**Number of studies included**	**Effect size (95%CI)**	**Homogeneity index, Q**	**Between-study variability, I** ^**2**^
Excluded studies did not give confounder-adjusted estimates	2 [19,31]	16	1.211 (0.975 to 1.505)	114.881, *P* <0.01	86.943
Excluded studies got a ‘moderate’ or ‘low’ risk of bias	3 [31,32,42]	15	1.178 (0.937 to 1.481)	144.866, *P* <0.01	90.336
Excluded studies did not reported RR or HR as effect size measurement	12 [3,19,31-34,36,39-43]	6	0.901 (0.622 to 1.305)	31.039, *P* <0.01	91.391

CI, confidence interval; HR, hazard ratio; RR, relative risk.

**Table 3:** **Details of subgroup analysis of pooled risk ratio (95%CI) for in-hospital mortality**

	**Number of studies included**	**Effect size (95%CI)**	**Homogeneity index, Q**	**Between-study variability, I** ^**2**^
Type of patient				
All ICU	7[2,3,19,32,35,38,42]	1.513 (1.123 to 2.039)	39.822, *P* <0.01	84.933
Sepsis and shock	2[4,39]	0.831 (0.203 to 3.413)	8.041, *P* = 0.005	87.563
Surgical	3[21,34,37]	1.174 (0.682 to 2.023)	6.793, *P* = 0.033	70.556
Trauma	3[31,36,43]	2.705 (0.815 to 8.973)	30.695, *P* <0.01	93.484
Other	3[33,40,41]	0.940 (0.417 to 2.116)	15.505, *P* <0.01	87.101
Outcome measurement				
Count data	2 [19,31]	3.969 (2.023 to 7.788)	28.936, *P* <0.01	96.544
OR	10 [2,3,32-34,36,39-42]	1.465 (1.049 to 2.045)	30.191, *P* <0.01	70.190
RR and HR	6 [4,21,35,37,38,43]	0.901 (0.622 to 1.305)	31.039, *P* <0.01	91.391
Adjustment				
Unadjusted	2 [19,31]	3.933 (2.107 to 7.343)	28.936, *P* <0.01	96.544
No propensity matched but multiple adjusted	9 [2,32-34,39-41,43]	1.358 (0.965 to 1.910)	25.626, *P* <0.01	68.782
Propensity score matched	7 [3,4,21,35,37,38,42]	1.089 (0.767 to 1.546)	60.523, *P* <0.01	90.086
Leukoreduced usage				
Not reported	11 [31-34,36,37,39-43]	1.851 (1.229 to 2.786)	86.863, *P* <0.01	88.488
<50%	3[2,4,19]	1.155 (0.583 to 2.290)	52.156, *P* <0.01	96.165
~76%	2 [3,35]	0.966 (0.412 to 2.267)	14.812, *P* <0.01	93.249
~100%	2 [21,38]	1.031 (0.452 to 2.352)	1.261, *P* = 0.26	20.696

CI, confidence interval; HR, hazard ratio; ICU, intensive care unit; RR, relative risk.

To assess factors responsible for heterogeneity of effect sizes across studies, we conducted meta-regression analyses. Six potential factors (publication year, mean admission Hb level, mean units transfused per patients, mean pretransfusion Hb level, mean admission acute physiology and chronic health evaluation II (APACHE II) score, and mean age of patients) were considered. The results suggested that publication year (coefficient −0.1056, *P* = 0.048) and mean age of patient (coefficient −0.0417, *P* = 0.002) had a significant influence. Table 4 summarizes the meta-regression analyses. In our meta-analysis, only two of the eighteen included studies had information on storage duration of RBCs [19,34], we thus could not conduct analysis on this topic.

**Table 4:** **Details of meta-regression of slope coefficients for in-hospital mortality**

	**Number of studies**	**Coefficient (95% ** **CI)**	***P*** **value**
Age of patient	18 [2-4,19,21,31-43]	−0.0417 (−0.0680 to −0.0154)	0.002
Publication year	18 [2-4,19,21,31-43]	−0.1056 (−0.2103 to −0.0009)	0.048
Admission Hb	9 [2-4,21,38-42]	−0.1848 (−0.4343 to 0.0648)	0.147
Units transfused	12 [2,3,19,21,31,32,35-37,42,43]	−0.1676 (−0.3856 to 0.0504)	0.132
Pretransfusion Hb	7 [2-4,21,34,42,43]	−0.2157 (−0.8588 to 0.4275)	0.511
APACHE II	8 [2-4,21,34,38,40,42]	0.0202 (−0.0569 to 0.0973)	0.607

APACHE II, acute physiology and chronic health evaluation II; CI, confidence interval; Hb, hemoglobulin.

### Publication bias

There was significant heterogeneity (*P* = 0.007) across studies. Visual inspection of the funnel plot (Figure S6 in Additional file 6) indicated the presence of a moderate publication bias. Begg’s test suggests that significant publication bias was not likely (*P* = 0.570). Furthermore, when looked for missing studies using the trim-and-fill random effects model, no studies needed to be imputed.

## Discussion

Whether RBC transfusion is linked to higher or lower mortality in critically ill patients has been a matter of debate for more than a decade. Our review was aimed to determine whether allogeneic RBC transfusion is beneficial or harmful for ICU patients, and clarify the nature of discrepancies among previous observation studies. The main findings are as follows.

- There is no consistent evidence linking RBC transfusion to in-hospital mortality in ICU patients
  The initially overall pooled results suggested that allogeneic blood transfusion was associated with an 11% to 85% increased risk for in-hospital mortality. However, after excluding studies with unadjusted estimates only [19,31] or studies with median overall quality [31,32,42], the association became null. Therefore, we could not draw a definitive conclusion. The results of sensitivity analyses suggested that such risk association may be attributable to studies without adjustment for confounders. In studies adjusted for confounders, studies that reached effect size by using the Cox regression model, and studies that implemented detailed information about leukodepleted blood measurement, we found that RBC transfusion did not increase risk of in-hospital death.
- There is no evidence indicating that RBC transfusion is associated with increased mortality in patients admitted to ICU for sepsis and shock, trauma and surgical reasons.
  Type of patients is an extremely important confounder for survival after RBC transfusion. Different types of patients have different responses to RBCs transfusion. For example, age of transfused RBCs could potentiate the risk association of volume of RBC transfused in trauma patients, but less so in other types of patients [44,45]. In our subgroup analysis, we separate enrolled studies into four groups according to patients’ population: all ICU admissions (seven studies), sepsis and shock (two studies), surgical (three studies), trauma (three studies), and others (three studies, one on patients with ALI or ARDS, one on patients with intracerebral hemorrhage, and one on patients with anemia and renal dysfunction). As presented in Table 3, the results suggested that there was no association between RBC transfusion and mortality in sepsis and shock, surgical, trauma and ‘other’ patients. The pooled overall effect estimate of seven studies on all ICU admissions was significant (RR 1.513, 95%CI 1.123 to 2.039). However, in these seven studies, we noticed that one study did not adjust for any confounders, therefore had a high risk of bias [19]. Actually, after excluding this study, we observed a 7% reduction but still significant pooled overall effect estimate (RR 1.406, 95% 1.016 to 1.945). In a large, multiple center study by Vincent et al., admission type of patients were elective surgery (41.4%), medical (32.6%), emergency surgery (16.6%), trauma (7.6%) and other (1.6%) [2]. In our analysis, we were unable to identify medical ICU patients as an independent subgroup. Literature on the safety of RBC transfusion in medical ICU patients was poor. In the SOAP study, medical admission was found to be an independent predictor for risk of 30-day mortality in ICU patients (RR 1.47, 95% 1.24 to 1.74) [35]. Further studies are needed to identify whether medical admissions are less tolerant of RBC transfusion than other patient populations.
- There is a lack of evidence on whether leukodepleted RBC transfusion could reduce in-hospital mortality in ICU patients.
  Immunomodulating effects of transfused RBCs have been suggested as potential cause of many adverse effects [46]. Leukocytes from donated blood may play a key role in immune system suppression; however, the exact mechanism is unclear [47]. Studies had reported a decrease in mortality rate among patients receiving prestorage leukoreduced blood compared to patients receiving nonfiltered blood [22,23]; however, a meta-analysis of 12 RCTs comparing leukodepleted and nonleukodepleted RBCs failed to identify an association between leukoreduction and mortality (OR 1.14, 95%CI, 0.89 to 1.45) [48]. Of the eighteen studies included in our review, only seven of them had information on percentage of patients receiving nonfiltered blood. Filtered RBCs were routinely used in two studies, Sakr et al. [21] suggested that RBC transfusion was independently associated with lower risk of mortality (RR 0.96, 95%CI, 0.95 to 0.98) while Engoren et al. [38] found no association. In our subgroup analyses, studies were divided into four groups according to the usage of leukoreduction. In a subset of 11 studies that did not report information on leukoreduction, the pooled effect estimate suggested that RBC transfusion was associated with higher mortality (RR 1.851, 95%CI, 1.229 to 2.786); however, the pooled effect estimates of other subgroups were not significant, regardless of the degree of implementation of leukodepleted blood (Table 3). Studies lacking information on leukoreduction had a higher risk of in-hospital mortality. Because of insufficient information, we cannot tell whether the use of leukodepleted blood could decrease mortality in transfused patients. Another possible explanation is that studies lacking leukoreduction information also had lower qualities, hence indicating high risk of bias. We recommend that further studies should take leukoreduction implementation into consideration.
- Study and statistical analysis methods are of considerable importance in explaining the association between RBC transfusion and in-hospital mortality.
  To model the association between RBC transfusion and in-hospital mortality, 10 of the 18 included studies used multivariable logistic regression, and reported OR as the outcome measurement. Pooled effect estimate of this subset of studies found that there is a risk association between RBC transfusion and mortality (RR 1.465, 95%CI, 1.049 to 2.045). In contrast, in five studies used Cox regression, there was no independent association between RBC transfusion and mortality (RR 0.901, 95%CI, 0.622 to 1.305). This may be because that selection of statistical model may have effect on the final result. As logistic regression did not take time-dependent variables into consideration, with increasing follow-up time, the logistic regression coefficients become less reliable [49]. For the same transfusion data, treating transfusion and living status as a time-dependent variable or not may result in different conclusions. Our review suggests that the statistical analysis method may cause disagreement between previous studies.
  Previous research revealed that older and more severely ill patients were more likely to have comorbidities after RBC transfusion [2,4]. The quality of a study relies on the completeness of data on possible confounders [34]. Patients’ admission characteristics and disease severity are two major confounders that can have an impact on transfused patients’ risk of death. Multivariable confounder-adjustment analyses and propensity score analyses are implemented in most of the studies to control imbalances between transfused and nontransfused groups. Under propensity matched groups, patients were matched by their probability to receive a transfusion, thus statistically reducing confounding bias. Previous findings revealed that propensity score analysis could produce estimates that were more precise and robust than regular logistic regression while there were seven or fewer events per confounding variables, but might produce unstable estimates when events per confounder were more than eight [50]. Most of our included studies reported multivariable confounder-adjusted estimates (Table 3), and the mostly used confounders in adjustments were patient characteristics at admission including age, gender and admission symptom score; seven studies used propensity score analyses. Subgroup analyses of the seven propensity score matched studies and the other nine no propensity score matched confounder-adjusted studies all showed no association between RBC transfusion and death (RR 1.089, 95%CI, 0.767 to 1.546; RR 1.358, 95%CI, 0.965 to 1.910 respectively), while the other two studies with unadjusted effect estimates only showed an increased risk (RR 3.933, 95%CI 2.107 to 7.343). This suggests that studies that did not fully adjust for confounders may overestimate the negative effects of RBC transfusion. This finding supported the view of Ruttinger et al. [34] that RBC transfusion may be only a surrogate marker for disease severity and is not causally related to mortality. Ruttinger’s study even suggested the pitfalls of propensity score and multivariate analyses; both cannot adjust for unobserved or unknown confounders, and not all factors that may influence transfusion practice had been collected. Ruttinger considered variables reflecting disease severity during ICU stay, and found that the number of RBC transfusion units was associated with increased mortality, and the association attenuated but persisted while only adjusted for admission variables. When controlling for variables of disease severity during ICU stay, this association vanished. Disease severity during ICU stay might be particularly important. We suggest that further research should take this into consideration. In our review, we performed meta-regression analyses to assess whether the mean number of units of blood transfused, mean admission Hb level and mean admission symptom score could explain disagreement between study results. Mean number of units of RBC transfused were reported in twelve studies, mean admission Hb level were reported in nine studies, mean APACHE II score were reported in eight studies, mean SOFA score were reported in four studies [2-4,42], and mean SAPS II [35] and mean APACHE III score [39] were reported in only one study, respectively. Results of meta-regression failed to identify significant association across studies for mean number of units of RBC transfused (coefficient −0.1676, 95%CI, −0.3865 to 0.0504), mean admission Hb level (coefficient −0.1848, 95%CI, −0.4343 to 0.0648) and mean APACHE II score (coefficient 0.0202, 95%CI, −0.0569 to 0.0973). In other words, these variables could not explain the disagreement across studies.
- Blood transfusion may decrease risk of mortality in older patients.
  The slope estimate was significant −0.0417 (95%CI, −0.0680 to −0.0154) (Figure S8 in Additional file 7), suggesting that with older age, transfused patients had better chances of survival. This is interesting since age has long been known as a predictor for worse outcomes including mortality. We speculate that this may be due to the following reasons. (1) Older patients and younger patients have different etiology of anemia. Older patients are more likely to have nutritional deficiency, chronic diseases and unexplained anemia [51]. Consequently, older patients and younger patients may differ in disease severity and clinical treatment. (2) Hb level may be a potential explanation. Using World Health Organization (WHO) definition of anemia (Hb level <13 g/dL in men and <12 g/dL in women), the corrected annual incidence of anemia increased with age [52], and the study found that even a ‘higher’ Hb level was associated with risk of death in older people [53]. Older patients may have different ‘optimal’ Hb concentrations, a ‘mildly low’ and ‘low-normal’ Hb level may be well tolerated in young patients, but not in older people [54,55].
  Literature on patient’s age, survival and blood transfusion is controversial in defining anemia in older people. Although we identified a significant association between mean age of patient and survival, there was no strong evidence to support this view. Further research could explore whether blood transfusion is more effective in improving survival in older patients.
- The debate on whether it is beneficial or harmful for RBC transfusion: articles tend to report lower risk effect estimates.
  The number of reports concerning such issues has grown fast in recent years, as shown in Figure S7 in Additional file 8. The meta-regression analysis produced a significant effect for publication year. Recent studies are more likely to report relativity lower risk ratios, indicating that RBC transfusion might have gradually become safer [56]. Safety concerns in RBC transfusion have emerged during the past two decades along with clinical practice changes [57]. The most important change in clinical practice is the recognition that the decision to transfuse should not be driven by a single Hb threshold, but by the physiologic state of the individual patient, presence of comorbidity, cardiopulmonary physiologic parameters, and evidence of blood loss [56,58]. In our meta-analysis, seven studies from the years 2002 to 2012 had reported average pretransfused Hb levels, and the mean Hb levels ranged from 6.6 g/dL to 8.6 g/dL. Meta-regression for pretransfusion Hb level failed to identify the significance for risk of mortality (coefficient −0.216, 95%CI −0.859 to 0.428).

### Limitations

It should be noted that the present meta-analysis had several limitations. First, all studies included in the meta-analysis were observational studies. Limited by study design, a causal relationship could not be determined. A convincible causal relationship needs further research in large RCTs. Second, limited by our recognition of exact mechanisms of transfusion-related complications, the potential confounders for death were not fully adjusted and may not all be included in our meta-analysis, this may have led to potential risk of bias. Third, the number of studies in subgroup and meta-regression analyses was relativity small, and therefore produced unstable effect estimates. Fourth, our study only focused on mortality within one month, while patients with death after one month of hospitalization may have high risk of transfusion. Finally, publication bias may still exist from unpublished studies, and publications in sources out of our literature search criteria.

## Conclusions

Our systematic review and meta-analysis summarized published literature on the relation between RBC transfusion and in-hospital mortality of ICU patients. We conclude that there is a lack of evidence to support the claim that ICU patients who receive RBC transfusion have an increased risk of in-hospital mortality. In studies that have adjusted for confounders, RBC transfusion did not increase the risk of in-hospital mortality in ICU patients. The review also suggests possible reasons to explain the discrepancies between previous studies. Our finding points to the need to identify risk factors for mortality in patients admitted to the medical ICU to account for all possible confounders for mortality. RCTs are warranted to provide convincible evidence of casual association.

## Key messages

- There was no consistent evidence to link RBC transfusion with mortality in ICU patients.
- In studies that had adjusted for confounders, RBC transfusion was not associated with in-hospital mortality in ICU patients.
- The discrepancies on risk of mortality and RBC transfusion between previous studies may be due to different types of patients, information on leukoreduction, study design and statistical methods, age of patients, and publication year.

## Additional files

Additional file 1: Figure S1. Risk of bias assessment. Additional file 2: Figure S2. Association between red blood cell transfusion and in-hospital mortality, grouped by type of patient. Additional file 3: Figure S3. Association between red blood cell transfusion and in-hospital mortality, grouped by outcome measurement. Additional file 4: Figure S4. Association between red blood cell transfusion and in-hospital mortality, grouped by adjustment for confounding. Additional file 5: Figure S5. Association between red blood cell transfusion and in-hospital mortality, grouped by leukoreduced usage. Additional file 6: Figure S6. Funnel plot for assessing publication bias of studies. Additional file 7: Figure S8. Meta-regression for mortality associated with mean age of patient. Additional file 8: Figure S7. Meta-regression for mortality associated with published year of article.

## Abbreviations

ALI: acute lung injury
APACHE: acute physiology and chronic health evaluation
ARDS: acute respiratory distress syndrome
CI: confidence interval
Hb: hemoglobin
HR: hazard ratio
ICU: intensive care unit
LOS: length of stay
OR: odds ratio
RBC: red blood cell
RCT: randomized controlled trial
RR: relative risk
SAPS II: simplified acute physiology score II
SOFA: sepsis-related organ failure assessment
TRALI: transfusion-related acute lung injury
