# Supplementary figures and images for: Association of red blood cell transfusion and in-hospital mortality in patients admitted to the intensive care unit: a systematic review and meta-analysis
Source: Crit Care. 2014 Nov 14;18(6):515. doi: 10.1186/s13054-014-0515-z (PMC4256753; doi:10.1186/s13054-014-0515-z)

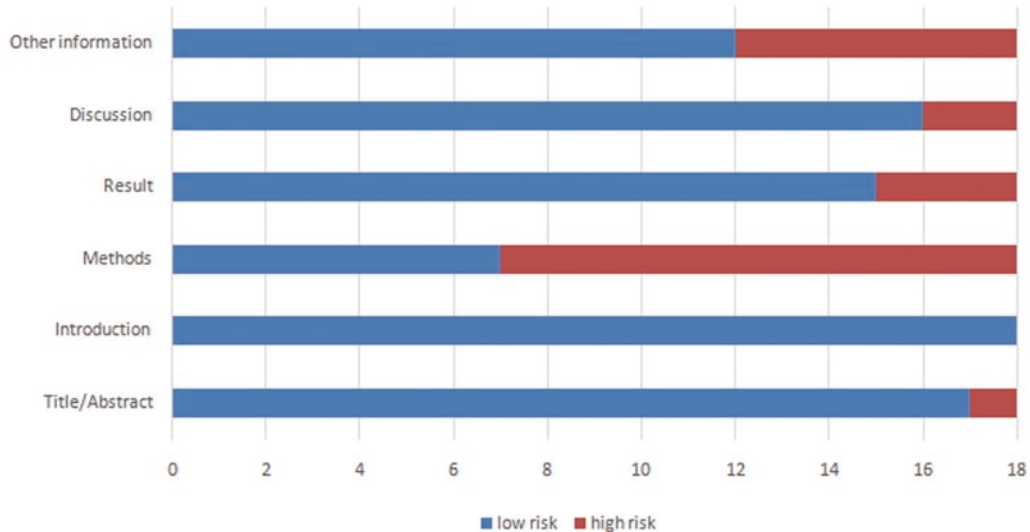

Supplement: Additional file 1: Figure S1. — Risk of bias assessment. [file 13054_2014_515_MOESM1_ESM.pdf]

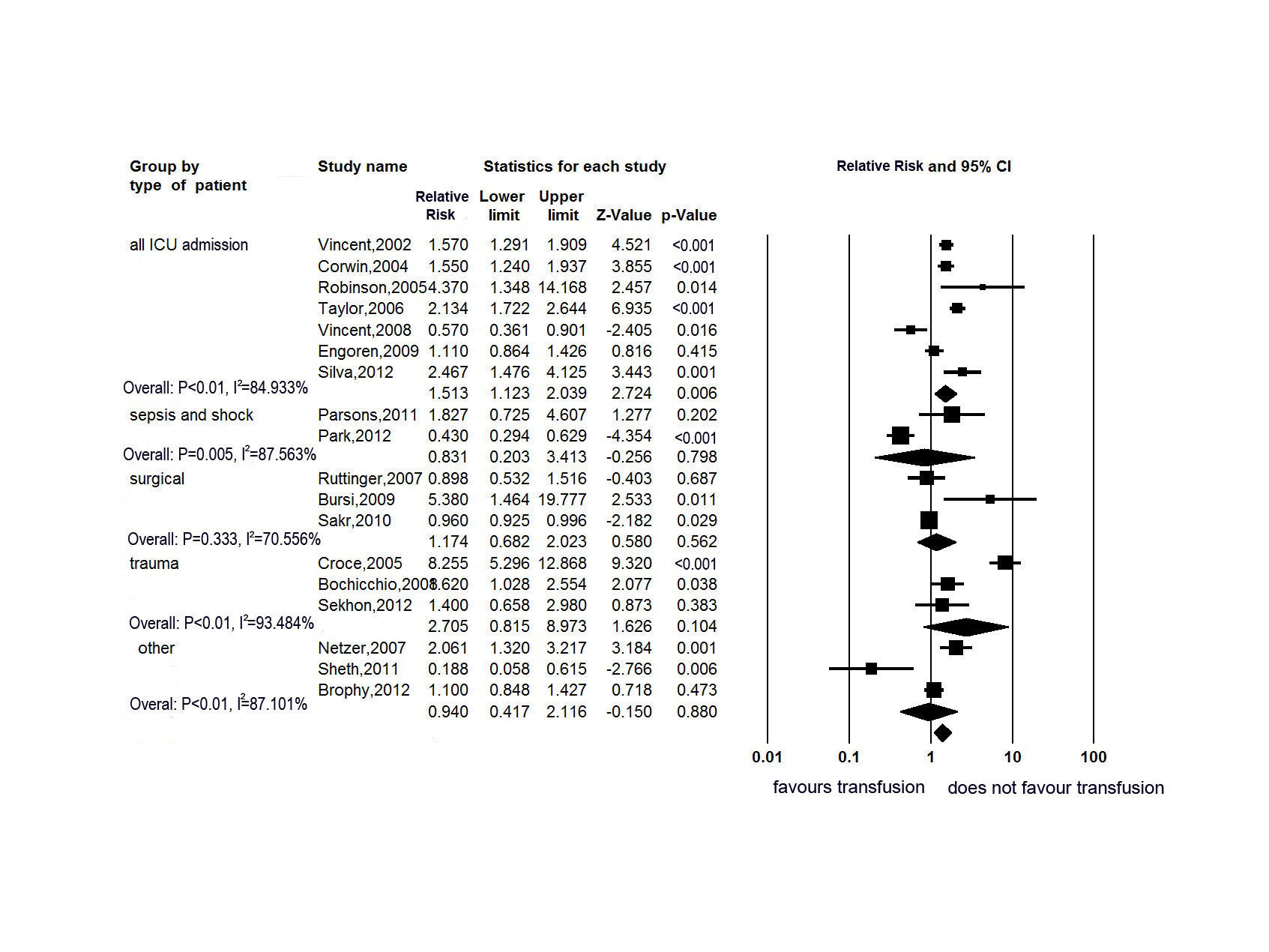

Supplement: Additional file 2: Figure S2. — Association between red blood cell transfusion and in-hospital mortality, grouped by type of patient. [file 13054_2014_515_MOESM2_ESM.jpeg]

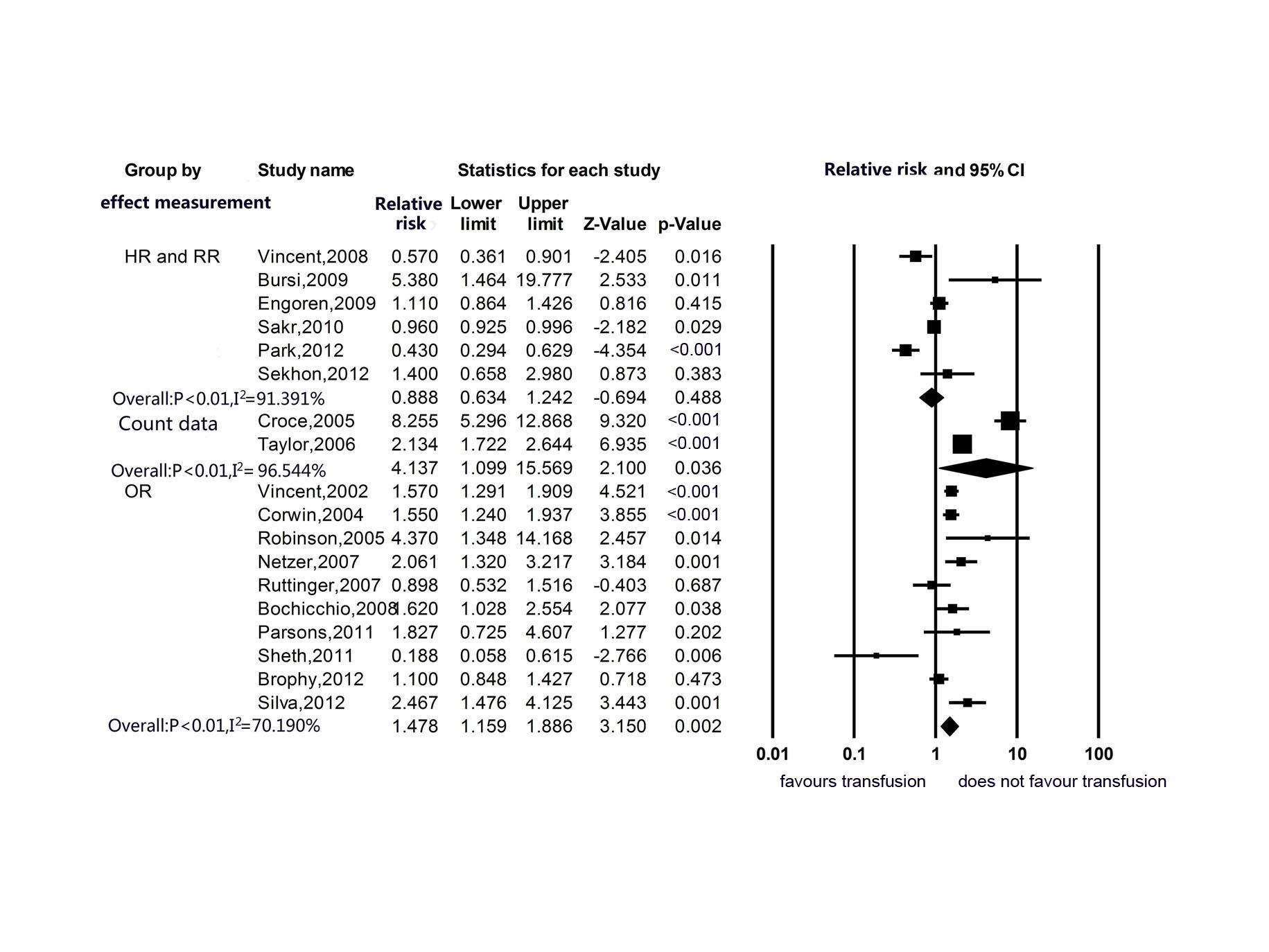

Supplement: Additional file 3: Figure S3. — Association between red blood cell transfusion and in-hospital mortality, grouped by outcome measurement. [file 13054_2014_515_MOESM3_ESM.jpeg]

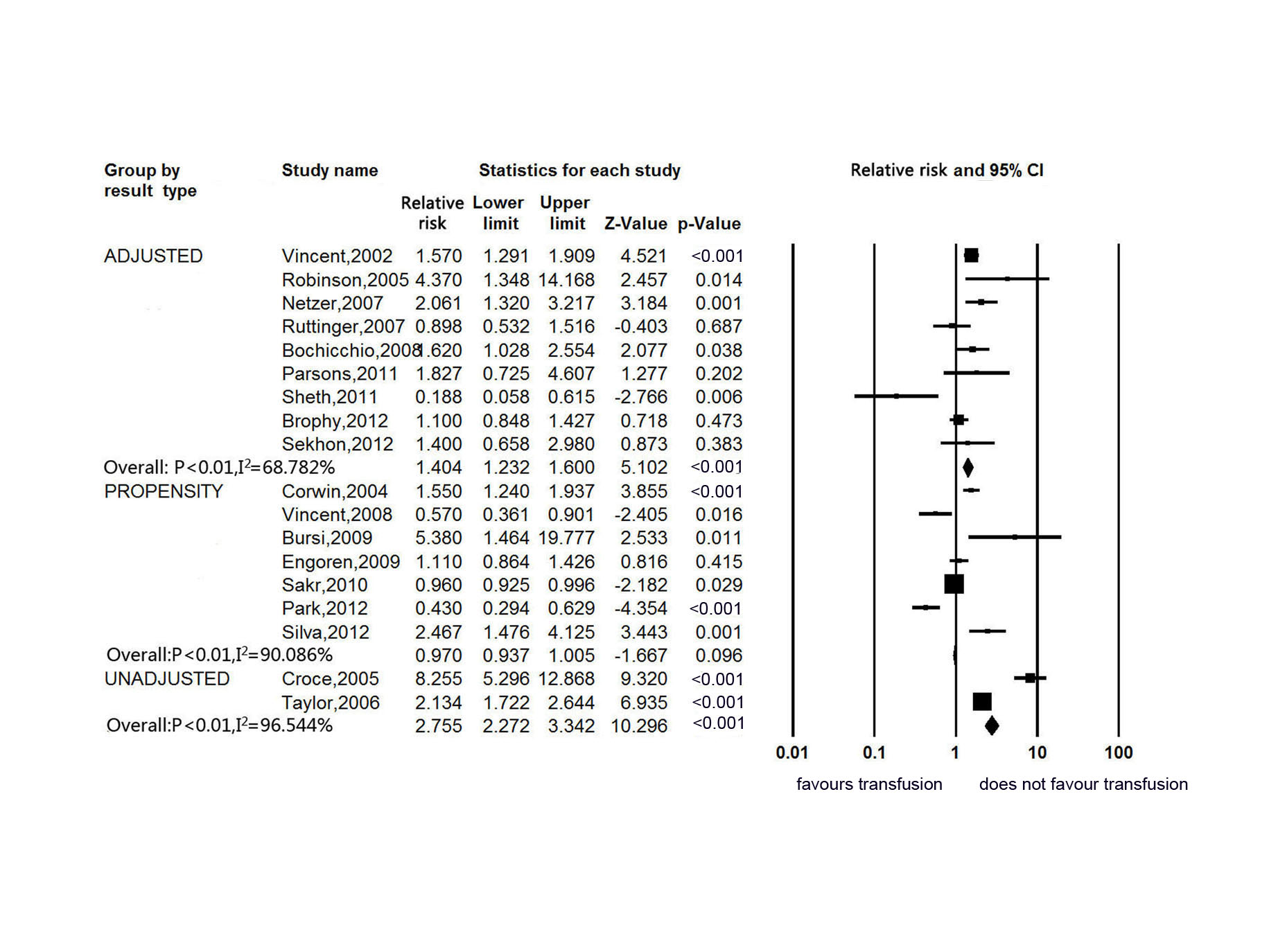

Supplement: Additional file 4: Figure S4. — Association between red blood cell transfusion and in-hospital mortality, grouped by adjustment for confounding. [file 13054_2014_515_MOESM4_ESM.jpeg]

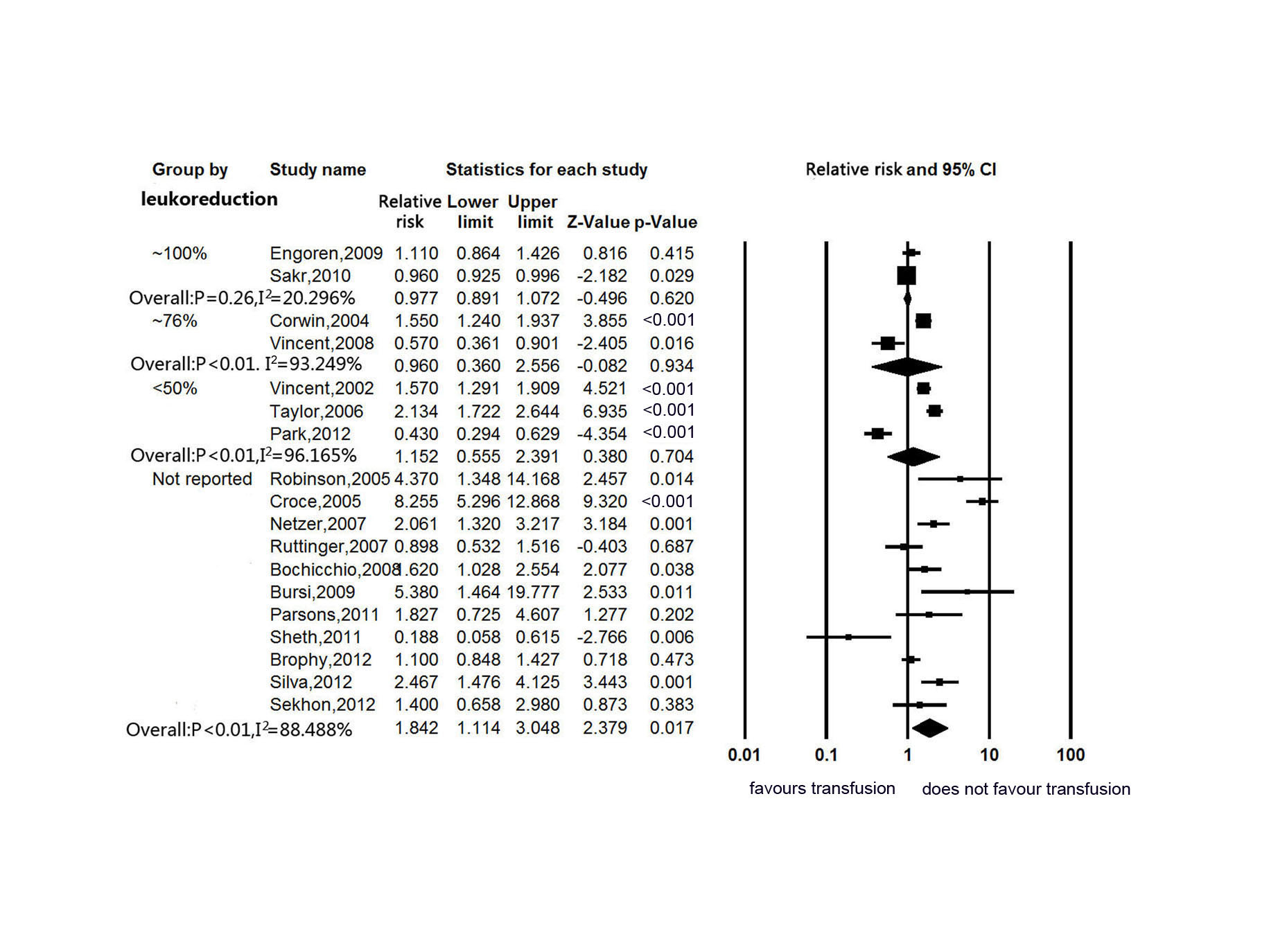

Supplement: Additional file 5: Figure S5. — Association between red blood cell transfusion and in-hospital mortality, grouped by leukoreduced usage. [file 13054_2014_515_MOESM5_ESM.jpeg]

Funnel Plot Standard Error by Log relative risk

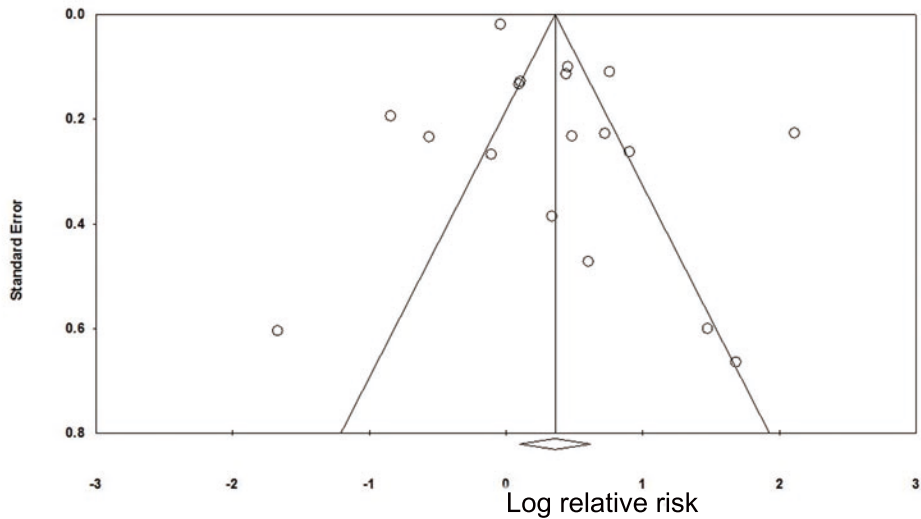

Supplement: Additional file 6: Figure S6. — Funnel plot for assessing publication bias of studies. [file 13054_2014_515_MOESM6_ESM.pdf]

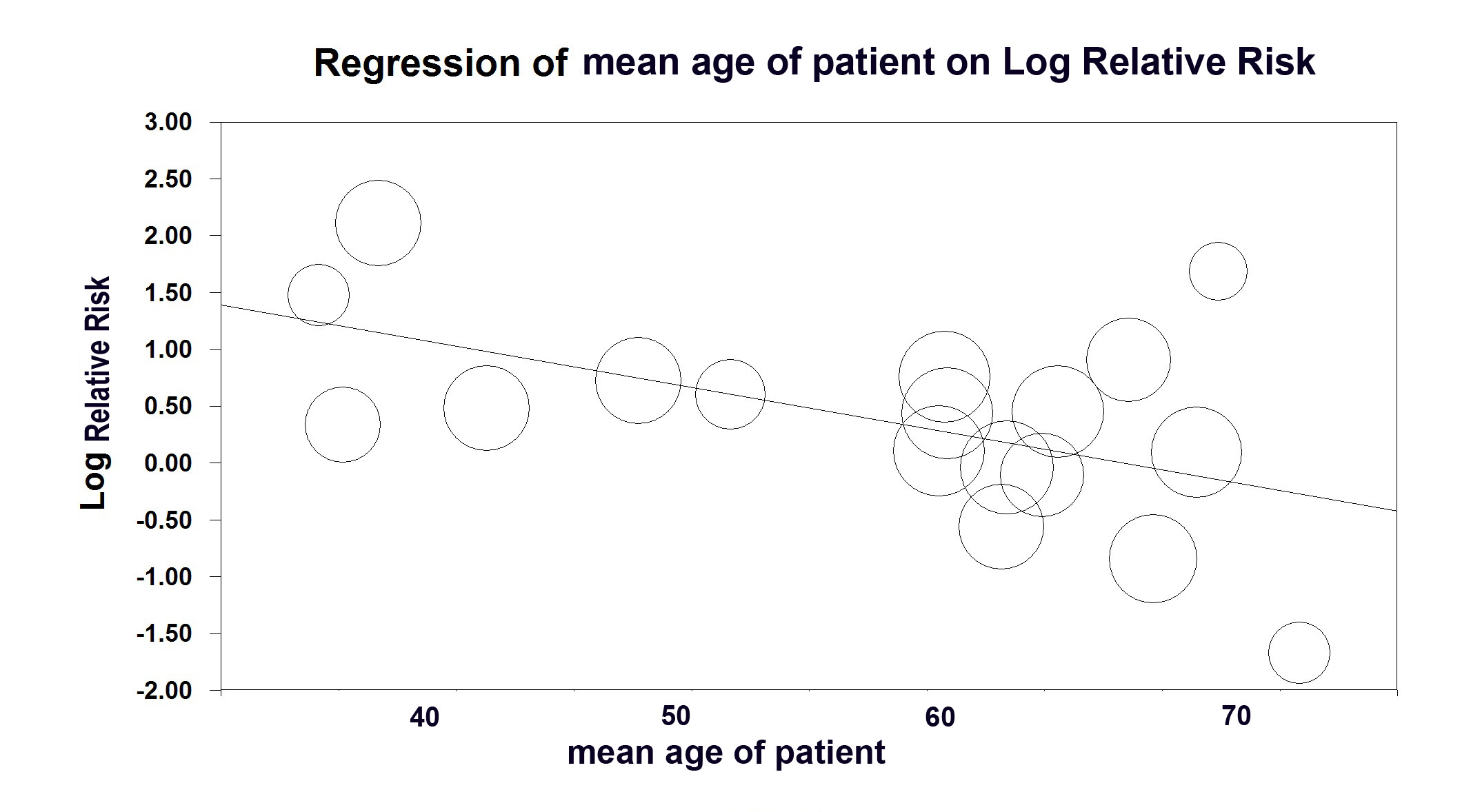

Supplement: Additional file 7: Figure S8. — Meta-regression for mortality associated with mean age of patient. [file 13054_2014_515_MOESM7_ESM.jpeg]

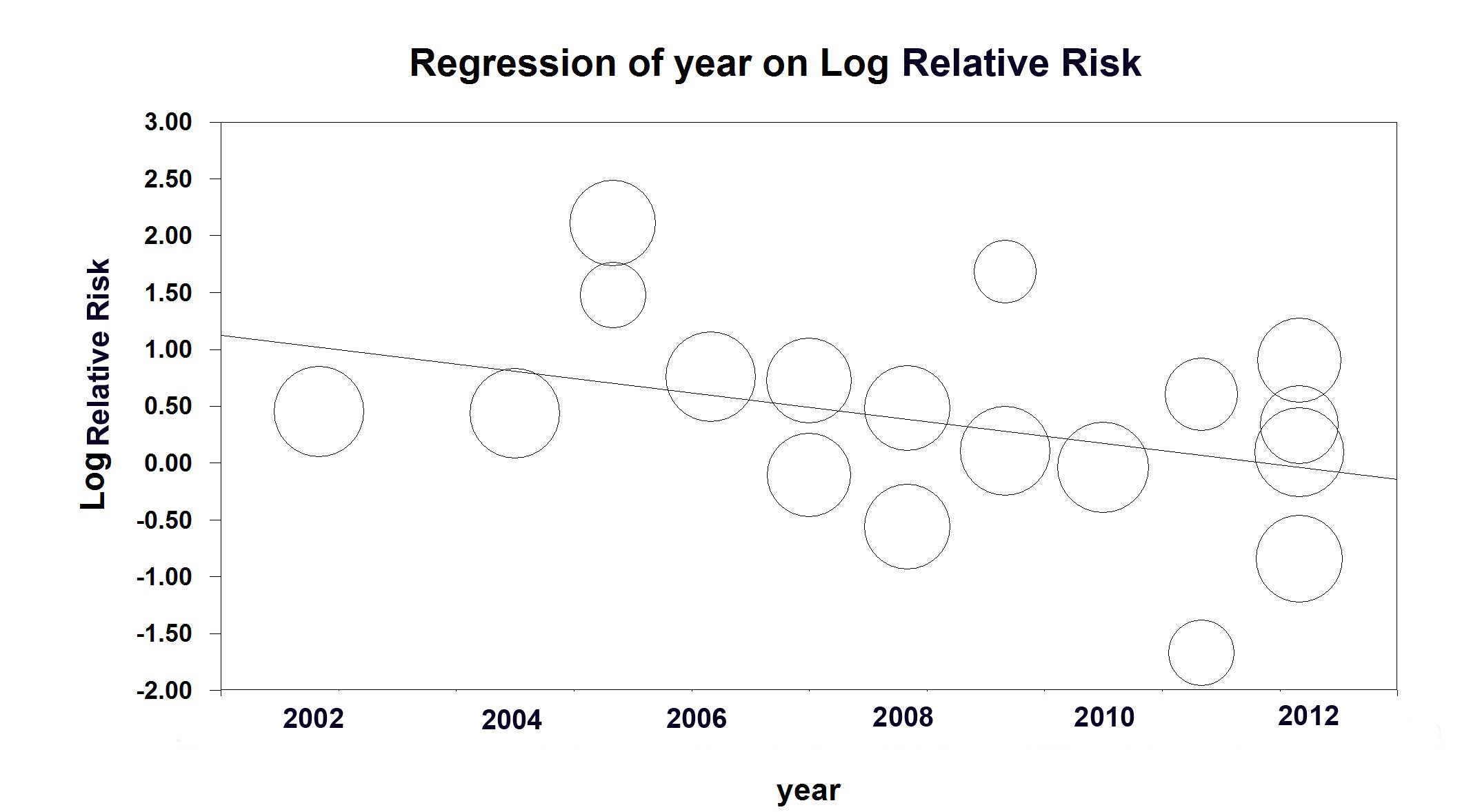

Supplement: Additional file 8: Figure S7. — Meta-regression for mortality associated with published year of article. [file 13054_2014_515_MOESM8_ESM.jpeg]
